# The classification of mRNA expression levels by the phosphorylation state of RNAPII CTD based on a combined genome-wide approach

**DOI:** 10.1186/1471-2164-12-516

**Published:** 2011-10-20

**Authors:** Jun Odawara, Akihito Harada, Tomohiko Yoshimi, Kazumitsu Maehara, Taro Tachibana, Seiji Okada, Koichi Akashi, Yasuyuki Ohkawa

**Affiliations:** 1 Faculty of Medicine Div. Epigenetics, Kyushu University, Fukuoka 812-8582, Japan; 2 Department of Medicine and Biosystemic Sciences, Kyushu University Graduate School of Medicine, Fukuoka 812-8582, Japan; 3 Department of Bioengineering, Graduate School of Engineering, Osaka City University, Osaka 558-8585, Japan

## Abstract

**Background:**

Cellular function is regulated by the balance of stringently regulated amounts of mRNA. Previous reports revealed that RNA polymerase II (RNAPII), which transcribes mRNA, can be classified into the pausing state and the active transcription state according to the phosphorylation state of RPB1, the catalytic subunit of RNAPII. However, genome-wide association between mRNA expression level and the phosphorylation state of RNAPII is unclear. While the functional importance of pausing genes is clear, such as in mouse Embryonic Stem cells for differentiation, understanding this association is critical for distinguishing pausing genes from active transcribing genes in expression profiling data, such as microarrays and RNAseq. Therefore, we examined the correlation between the phosphorylation of RNAPII and mRNA expression levels using a combined analysis by ChIPseq and RNAseq.

**Results:**

We first performed a precise quantitative measurement of mRNA by performing an optimized calculation in RNAseq. We then visualized the recruitment of various phosphorylated RNAPIIs, such as Ser2P and Ser5P. A combined analysis using optimized RNAseq and ChIPseq for phosphorylated RNAPII revealed that mRNA levels correlate with the various phosphorylation states of RNAPII.

**Conclusions:**

We demonstrated that the amount of mRNA is precisely reflected by the phased phosphorylation of Ser2 and Ser5. In particular, even the most "pausing" genes, for which only Ser5 is phosphorylated, were detectable at a certain level of mRNA. Our analysis indicated that the complexity of quantitative regulation of mRNA levels could be classified into three categories according to the phosphorylation state of RNAPII.

## Background

Cellular function is accomplished by the accurate, regulated transcription of genes in the genome. The quantity of transcribed mRNA of protein-coding genes varies, and the regulation of transcription is carried out by a wide variety of nuclear factors on the chromatin structure. One of the key regulatory mechanisms is the control of the activation of RNA polymerase II (RNAPII) [1].

RNAPII transcribes all protein-coding genes and many non-coding genes, and the activity of RNAPII correlates with the phosphorylation state of RPB1, the large catalytic subunit of RNAPII [2]. RPB1 has an unusual C-terminal domain (CTD) that consists of repeats of the heptapeptide consensus sequence N-Tyr1-Ser2-Pro3-Thr4-Ser5-Pro6-Ser7-C, of which there are 52 copies in mammals [3]. The amino acids in these repeats are potential targets for modification, such as phosphorylation and glycosylation. During transcriptional regulation, free hypophosphorylated RNAPII is recruited to gene promoters. RNAPII's escape from the promoter requires TFIIH, a general transcription factor that mediates phosphorylation of CTD Ser5 [4]. After promoter escape, RNAPII can move downstream of the transcription start site (TSS) [5]; however, pausing factors, such as NELF and DSIF, prevent productive elongation of mRNA [6]. This phenomenon is known as promoter proximal pausing [7]. Productive elongation of mRNA is coupled with phosphorylation of the CTD Ser2 residue [8]. The influence of promoter proximal pausing of RNAPII may contribute to the control of gene expression levels [9-11]. It is possible that full length mRNA cannot be detected because of pausing, and that a wide variety of expression levels, including high expression, are regulated by pause site entry and escape of RNAPII [7]. Recent studies revealed that RNAPII could bind to the promoter region of inactive genes in human fibroblasts [9], as well as in ES cells [10]. Additionally, in mouse ES cells, Ser5 phosphorylated and Ser2 unphosphorylated RNAPII accumulates around the TSSs in bivalent genes [11]. These genes, as differentiation markers, can be detected at low levels, despite their association with pluripotency [12]. High throughput sequencing technology and cDNA analysis have emerged as revolutionary tools in recent years, but whether these sequencing data come from active transcription or pausing state genes, and the genome-wide phosphorylation status of RNAPII in vivo, have not been studied. Several genes in which RNAPII is in the pausing state play key role in differentiation [12]; therefore, understanding the correlation of RNAseq and RNAPII phosphorylation state is very important. To evaluate the phosphorylation status of RNAPII for all genes identified with RNAseq, we have to exclude free RNAPII, in which Ser2 and Ser5 residues are not phosphorylated, and distinguish actively transcribed genes, for which both of Ser2 and Ser5 residues are phosphorylated, from pausing state genes, for which Ser5 residues are only phosphorylated. Evaluation of the relationship between the phosphorylation state of RNAPII and mRNA expression level will permit the identification of those genes that are actively transcribed and those that are pausing.

A variety of techniques have been developed to quantify and analyze gene expression levels, such as northern blotting, RT-qPCR, SAGE, and microarrays. Recently emerged deep sequencers enable the analysis of mRNA expression with much less bias compared with previous technologies, by reading tens of millions of tags in a single run (RNAseq) [13]. RNAseq can clarify the amount of previously identified transcripts [14], identify novel transcripts [15], and analyze tissue-specific alternative splicing [16]. RNAseq is 1,000 times more sensitive than microarrays for quantifying transcripts, and appears to be the best currently available tool for the evaluation of mRNA [17]. However, RNAseq has its own limitations. One such limitation is the need for reference sequences. The deep sequencer examines 25-200 bp short fragments, unlike previous technologies, and sequences tens of millions of fragments in a single run. These fragments, also known as 'reads', are mapped to a reference transcriptome to identify gene expression. However, because the transcriptomes are incomplete, even for well-studied species such as human and mouse, analysis of RNAseq data is restricted by the reference sequence, and requires another calculation to identify novel transcripts. TopHat [18] does not depend on a reference transcriptome, and provided a new way to evaluate novel transcripts, including new splicing sites. In addition, Cufflinks [19] can map reads to a reference genome and identify all transcripts quantitatively per kilobase of nucleotides and considers splicing. The weak point of quantification by these mapping techniques is the comparatively short sequence tag used to map to the reference genome. Success in mapping a sequence depends on the structure of the mRNA; it may have homologs that have a common structure, which may introduce bias to the statistical results. Therefore, to overcome these biases, it is necessary to use not only unique information, where one tag is mapped to one genomic region, but also multiple hit information, where one tag is mapped to two or more genomic regions. In TopHat the parameter 'Max multihits' controls how many regions one tag is allowed to map to, thereby optimizing mapping efficiency. However, a detailed evaluation of the influence of this parameter setting on the identification of mRNA has not been performed.

Thus, we used a deep sequencer to clarify how various mRNA expression levels are controlled, by analyzing the regulation of RNAPII through CTD phosphorylation. We categorized gene expression by identifying the phosphorylation control of RNAPII for all genes. In addition, by combining these data with genome-wide gene expression data that were obtained from RNAseq using the optimized 'Max multihits' parameter, we clarified the correlation between various mRNA expressions and RNAPII phosphorylation.

## Results and Discussion

### The accuracy of RNASeq is improved by permitting a small number of 'multihits'

**Figure 1:**
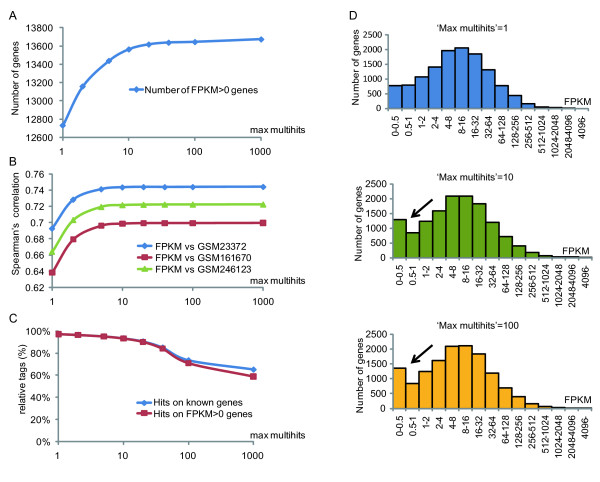
**Plots to assess the effect of the parameter 'Max multihits' (X-axis)**. 'Max multihits' is a TopHat parameter that indicates how many times one tag is permitted to be aligned. (a) Number of genes found by RNAseq (Y-axis) against 'Max multihits'. (b) Spearman's correlation between microarrays (GSM23372, GSM161670, and GSM246123) and RNAseq. (c) Y-axis indicates how many splicing sites found in TopHat were included in known genes or FPKM > 0 genes. (d) Histogram showing the distribution of FPKM. As the number of permitted 'Max multihits' increased, the quantity of genes with small FPKM values increased (black arrows).

To understand the transcriptional regulation mechanisms mediated by RNAPII, it was necessary to evaluate mRNA expression as accurately and quantitatively as possible. Many mRNAs possess high sequence similarity to their homologs in the genome, and when an increased number of 'Max multihits', one of parameters of TopHat, are permitted, we predicted that a greater number of genes would be identified. Figure 1A shows RNAseq analysis data in Hela cells, a popular human cell line for which there are abundant existing microarray data and reference sequence information, and shows the number of FPKM > 0 genes (FPKM: fragments per kilobase of exon per million fragments mapped, calculated by both TopHat and Cufflinks) identified with increasing Max multihits. It is clear that higher numbers of genes are identified when 'Max multihits' is increased, which means the sequences of two or more genes are taken forward for analysis. Figure 1B shows Spearman's correlation coefficient plotted against increasing 'Max multihits' for three previously published expression microarray data sets: GSM23372 [20], GSM161670 [21], and GSM246123 [22], and the value of FPKM. Though they are independent data sets, they show a high level of correlation that is not inferior compared with previously published analyses [17]. The correlation coefficient shows a tendency to rise with increasing 'Max multihits'. However, when 'Max multihits' becomes ten or more, both the number of identified genes (Figure 1A) and the correlation coefficient between the expression microarray and RNAseq (Figure 1B) hardly changes. This takes into account the fact that if a moderate multihit limit were not allowed, homologous sites would be excluded from RNAseq analysis. Conversely, increasing multihits too far introduces the danger of counting genes that were not originally expressed. To identify any negative influences of increasing 'Max multihits', we analyzed RNAseq data taking into account splicing. TopHat performs an alignment to the genome by dividing the cDNA sequence, and when it matches the genome, TopHat presumes that place to be a splice junction. Figure 1C shows the number of splice junctions found by TopHat in known genes or FPKM > 0 genes plotted against increasing 'Max multihits'. The proportion of splice sites found in genes other than already-known genes, or FPKM > 0 genes, increases as 'Max multihits' increases. In addition, we examined the influence of increasing Max multihits (1, 10, or 100 permitted) on the number of genes present in a range of FPKM values (15 groups, FPKM). The figure shows that the only significant change was an increase in the number of genes in the FPKM 0-0.5 group. From these results, we determined that the optimum setting for 'Max multihits' was 10. This identified the maximum number of genes, saturated the correlation between RNAseq and past expression microarray data sets, and reduced the possibility false positives. The accuracy of this optimization was reinforced by performing RT-PCR on genes with a small FPKM value (0.05-1.97) when 'Max multihits' was set to 10, and confirming the mRNA expression (Additional File 1, Figure S1).

### The distribution of phosphorylated RNAPII

**Figure 2:**
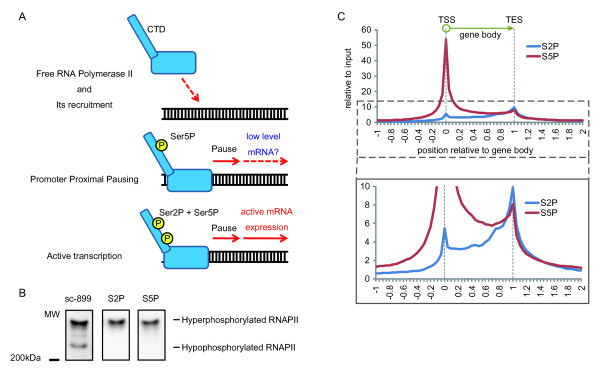
**Generation and assessment of anti-phosphorylated Ser2/Ser5 RNAPII antibodies**. (a) RNAPII was categorized by the phosphorylation status of its C-terminal domain (CTD). (a) Free RNAPII was hypophosphorylated in its CTD residues, and recruited to the promoter, where TFIIH phosphorylates Ser5 residues. Ser5-phosphorylated RNAPII progresses to the pausing site, but it is halted by negative elongation factors. Phosphorylation of Ser2 allows active transcription. Recent evidence suggests that several pausing genes produce full-length transcripts at low levels. (b) Reactivity of different RNAPII antibodies against hyper- and hypophosphorylated RNAP2 was assessed by immunoblotting. (c) The average Ser2/Ser5 phosphorylated RNAP2 enrichment tags per one gene from ChIPseq data, normalized to input by Peakseq, were summed according to their shifted positions, with the definition that gene length was from 0 to 1. Where 0 represents the transcription start site and 1 represents the transcription end site.

It has been reported that RNAPII transcribing activity correlates with the phosphorylation status of RNAPII(Figure 2A) [7]. Systematic and genome wide evaluation for the separation of pausing and active transcription states of RNAPII has not been done with the most frequently used antibodies, which recognize the phosphorylation state of RNAPII. Some of these antibodies were characterized as having limited activity to specific phosphorylation states [23]. Thus, we produced antibodies that specifically recognized Ser-2-phosphorylated (Ser2P) or Ser-5-phosphorylated (Ser5P) RNAPII to analyze the relationship between transcriptional levels and RNAPII phosphorylation for ChIPseq. Stock et al. (2007) showed that RNAPII could be classified into hyperphosphorylated RNAPII, in which Ser2 and/or Ser5 residues of the C-terminal domain are phosphorylated, and hypophosphorylated RNAPII, in which neither were phosphorylated, by western blotting analysis. Figure 2B shows that, in whole cell lysate of Hela cells, the antibodies to Ser2P and Ser5P specifically recognized hyperphosphorylated RNAPII, while antibody sc-899, which was raised against the N-terminus of RNAPII, recognized RNAPII regardless of its phosphorylation status. Monoclonal antibodies against the CTD can be influenced by the context of the antigen and the phosphorylation status of surrounding peptides [23]. Thus, to confirm the specificity of our antibodies to phosphorylation of Ser2 or Ser5, ELISA was performed using the CTD repetitive sequence (N-Tyr-Ser-Pro-Thr-Ser-Pro-Ser-C) phosphorylated at Ser2, Ser5, and Ser7 (singly or in combination) as an antigen (Table 1). The antibody to Ser2P recognized only Ser2P of RNAPII and was hardly influenced by phosphorylation of surrounding Ser5 or Ser7. By contrast, the antibody against Ser5P was influenced by Ser7P on the downstream side, but not by Ser2P. Thus, our antibodies specifically recognized Ser2P and Ser5P of the CTD under suitable conditions. Next, to evaluate the distribution of RNAPII on the genome, ChIPseq (which uses chromatin immunoprecipitation (ChIP) and deep sequencing to analyze DNA-protein interactions) was performed with these antibodies. All genes containing a region that coincided with part of a peak that was judged to be a significant peak under the condition of false discovery rate (FDR) < 0.05 and P-value < 0.05 by Peakseq [24] were selected. For these genes, using a previous model of analysis [25], detected gene body tags of ChIPseq were confirmed for all tags, and the total number of tags relative to input per one gene was calculated. Figure 2C shows the antibody to Ser5P exclusively detects high peaks around the transcription start site (TSS), and the antibody to Ser2P detects high peaks over the entire gene body, particularly around the transcription end site (TES). These results agreed with those of a previous study [12]. While relative tag count per one gene was almost the same for anti-Ser2P and anti-Ser5P around the TES, the total of relative tag count of Ser5P around the TSS was about ten times higher than that of Ser2P. These results indicate that these antibodies could distinguish between hyperphosphorylated RNAPII in the promoter proximal pausing state (Ser5P+, Ser2P-) and the active transcription state (Ser5P+, Ser2P+), and they also indicate that RNAPII performs genome wide movement, as previously described [7].

**Table 1:** The composition of the CTD and the reactivity of each antibody

CTD	Peptide	anti-Ser2P	anti-Ser5P
unmodified	SPTSPSYSPTSPSYSPTSPS	-	-
Ser2P	SPTSPSYSphPTSPSYSPTSPS	++	-
Ser5P	SPTSPSYSPTSphPSYSPTSPS	-	++
Ser7P	SPTSPSYSPTSPSphYSPTSPS	-	-
Ser2PSer5P	SPTSPSYSphPTSphPSYSPTSPS	+	++
Ser5PSer7P	SPTSPSYSPTSphPSphYSPTSPS	-	-
Ser7PSer2P	SPTSPSphYSphPTSPSYSPTSPS	++	-

### RNASeq can detect the expression of most genes, even in the "pausing" genes

**Figure 3:**
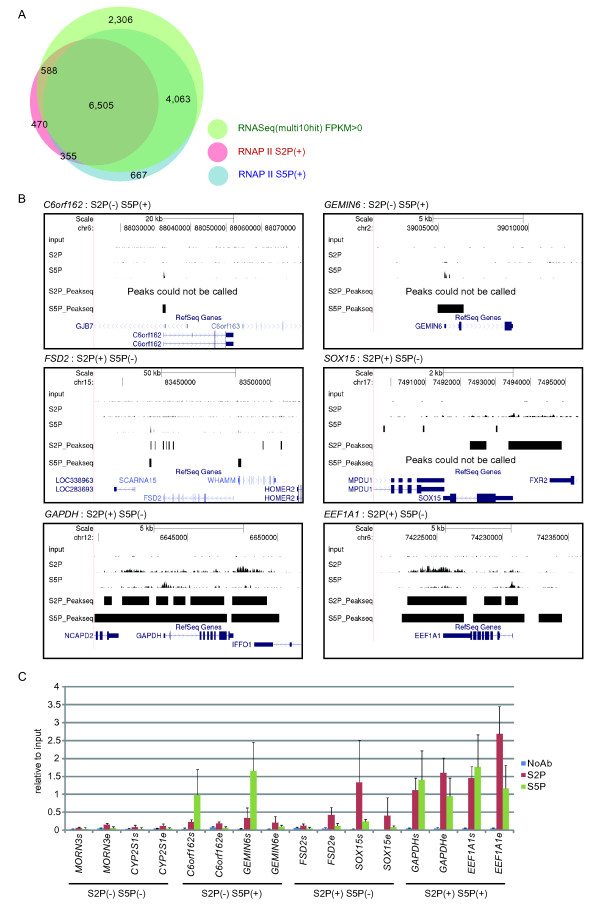
**Expressed genes were categorized by the phosphorylation state of RNAPII**. (a) A Venn diagram summarizing the overlap between FPKM > 0 genes, Ser2P-RNAPII positive genes, and Ser5P-RNAPII positive genes. We defined the presence of RNAPII to be positive if the region of the peak found by Peakseq overlapped the gene body. (b) Tags found by ChIPseq and peaks found by Peakseq (P-value < 0.05, FDR < 0.05) were visualized on the UCSC Genome Browser. (c) The abundance of different phosphorylated forms of RNAPII at Ser2P and/or Ser5P positive genes assessed by ChIP and qPCR at the coding region near the TSS and TES.

In embryonic stem (ES) cells, Ser5-phosphorylated RNAPII already exists on bivalent genes and pauses at the promoter proximal region [12]. This may suggest that pausing genes have a key role at the beginning of differentiation. However, to date, no report has established which phosphorylation state of RNAPII corresponds to the gene expression identified by RNAseq. How many genes defined as 'pausing' genes actually express mRNA and how much mRNA from 'pausing' genes is transcribed is also still unclear. To examine how many active transcribing genes and pausing genes were identified by RNAseq, we constructed a Venn diagram (Figure 3A, Additional File 2, Figure S2) from ChIPseq and RNAseq data. When the parameter of 'Max multihits' was expanded, the genes detected only by RNAseq, but not by RNAseq and ChIPseq, increased (Additional File 2, Figure S2). Increasing multihits too far introduces the risk of counting silent genes; therefore, we used the optimized parameter 'Max multihits = 10', and used genes with FPKM > 0. Peaks that conformed to P-value < 0.05 and FDR < 0.05 were assumed to be positive peaks using Peakseq [24], a Peakcaller in ChIPseq. Although an RNAPII that pauses around the promoter could be bound only to the region upstream of the TSS, a previous report demonstrated that a pausing RNAPII extends over the coding region [12]. Therefore, we defined that the association of Ser2- and/or Ser5-phosphorylated RNAPII with a gene was positive only when a part of the positive peak corresponded to a part of the gene body, to prevent the risk that other genes were mistakenly selected from very gene-dense genomic regions. All 23,821 human genes defined by RefSeq were evaluated. As a result, 14,954 genes (62.7%) of all genes had an FPKM > 0 in RNAseq or were judged to be positive for Ser2- and/or Ser5-phosphorylated RNAPII in ChIPseq.

Among 7,918 genes in which Ser2P is positive, 6,860 genes (87%) were assumed to be Ser5P positive too. On the other hand, among the 11,590 genes in which Ser5P is positive, 6,860 genes (59%) are assumed to be Ser2P positive. This result indicates that Ser5 and Ser2 of RNAPII have to be sequentially phosphorylated for active transcription, as described previously [26]. However, 1,058 genes (13%) are Ser2P positive only. When these genes are observed in the UCSC genome browser (University of California, Santa Cruz) (Figure 3B), Ser2P single positive genes appear in the comparatively gene-dense areas. Moreover, when the ChIP-qPCR data were verified (Figure 3C), for instance, for *SOX15*, which is judged to be a Ser2P single positive, more Ser2P was identified around the TSS than around the TES, although the amount of Ser2P did gradually increase towards the TES (Figure 2C). These results suggest that Ser2P single positive genes are false positives caused by the influence of surrounding genes or non-annotated transcripts in these regions. RNAPII with an unphosphorylated CTD is first recruited to a promoter region and is then released when its Ser5 is phosphorylated. Active transcription is then initiated when Ser2 is phosphorylated; however, RNAPII keeps running until its termination, even if transcription ends [27]. This results in the deterioration of the resolution of ChIPseq and it may be one factor that causes false positives in gene-dense areas. To overcome this limitation, we set a criterion in which we scored a peak as positive only when the peak extended over the gene body. Although this may affect the detection of RNAPII that is in the state of promoter proximal pausing, Ser5-phosphorylated RNAPII that is pausing around the TSS seems to be sufficiently detected when using this condition (Figure 3A-C).

Interestingly, RNAseq detected highly expressed genes not only in the state of active transcription (Ser5P+, Ser2P+), but also in the state of promoter proximal pausing (Ser5P+, Ser2P-), in the majority of FPKM > 0 genes. These results indicated that the phosphorylation of Ser5 and Ser2 correlates with gene expression in two stages. It also indicates that RNAseq, because of its high sensitivity, disregards the background epigenetic expression adjustment machinery associated with RNAPII phosphorylation. Some of the differentiation markers that were Ser5P single positive showed low mRNA expression in mouse embryonic stem cells [12]. However, we should take note of the expression of differentiation markers, as interpreted by RNAseq, in stem cells, because some of these genes could be identified as a result of RNAseq's high sensitivity.

Among the 13,462 genes which RNAseq judged to have an FPKM value > 0, 11,156 genes (83%) are Ser2P and/or Ser5P positive. The remaining 2,306 genes (17%) with FPKM > 0 in RNAseq, were identified as neither Ser2P nor Ser5P in ChIPseq. Among 12,648 genes which ChIPseq judged to be Ser2P and/or Ser5P positive, 11,156 genes (88%) were FPKM > 0 genes in RNAseq.

To further investigate functional relationship among pausing/active genes and gene functions, we analyzed significant associations using Gene ontology [28] and Fishers' exact test(Additional File 3, Table S1). Hundreds of GO terms were calculated to be significant for active genes, and some of GO terms associated with mitochondorial genes were judged to be significant for pausing genes. Neither calculation seemed to give significant enrichment of specific genes, except for housekeepking genes.

### Gene expression levels reflect the level of phosphorylation of RNAPII

**Figure 4:**
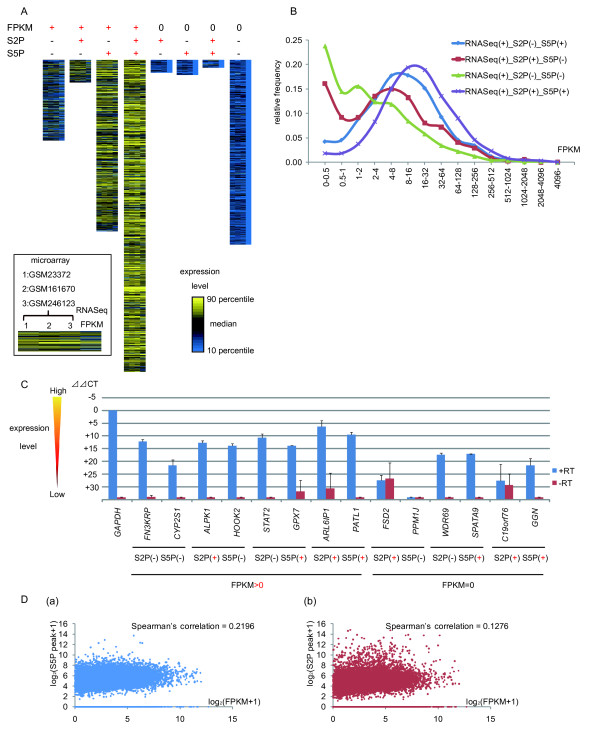
**mRNA expression level correlates with the phosphorylation of RNAPII**. (a) Expressions of genes assessed by expression microarray (left: GSM23372, middle left: GSM 161670, middle right: GSM246123) and RNAseq (right). Each column indicated classified genes by FPKM (= 0, or > 0) and RNAPII Ser2P and Ser5P binding state. Only those genes that had microarray expression data were analyzed; thus, there were 1,225/2,306 (53%) FPKM > 0/Ser2P(-)/Ser5P(-) genes, 348/588 (59%) FPKM > 0/Ser2P(+)/Ser5P(-) genes, 2,601/4,063 (64%) FPKM > 0/Ser2P(-)/Ser5P(+) genes, 4,727/6,505 (73%) FPKM > 0/Ser2P(+)/Ser5P(+) genes, 198/470 (42%) FPKM = 0/Ser2P(+)/Ser5P(-) genes, 232/667 (35%) FPKM = 0/Ser2P(-)/Ser5P(+) genes, 122/355 (34%) FPKM = 0/Ser2P(+)/Ser5P(+) genes, and 2,803 FPKM = 0/Ser2P(-)/Ser5P(-) genes available for analysis. Color key indicates gene expression value, yellow: over 90 percentile, black: median, blue: 10 percentile. The number of genes that were assessed was restricted by the microarray platform. (b) Histogram showing how many genes exist for each FPKM value. Y-axis indicates relative frequency in each category (RNAseq(+), Ser2P(+ or -), Ser5P(+ or -). Significant peak shift of the distribution of gene expression is shown according to each category. (c) Transcripts derived from genes that are categorized by Figure 3A were quantified by qPCR. The expressions of not only RNAseq(+) genes, but also some RNAseq(-) ChIPseq(+) genes are confirmed. (d) Assessment of the correlation between peak height of ChIPseq and FPKM value of RNAseq. Peak height for each gene was calculated by extracting the highest one that existed in the coding region. There is no obvious correlation.

To assess how much the phosphorylation of the CTD of RNAPII correlates with genome-wide gene expression, we examined the amount of mRNA expression by RNAseq in each part of the Venn diagram (Figure 3A). To validate results in independent data, expression microarray data sets, GSM23372 [20], GSM161670 [21], and GSM246123 [22], were evaluated at the same time. A heatmap (Figure 4A) and a histogram (Figure 4B) were then produced using the data of RNAseq and the expression microarrays. The amount of mRNA expression was the highest in the area representing Ser2P and Ser5P double positive by ChIPseq, and decreased in the order of Ser5P single positive, Ser2P single positive, and Ser2P/Ser5P double negative, for FPKM > 0 genes (Figure 4A, B). However, in genes judged to be FPKM = 0, no high levels of expression were observed in any of the three expression microarray data sets. In addition, we extracted genes at random and performed qPCR to validate the data (Figure 4C). qPCR generated similar results to Figure 4A, and expression was confirmed for all of the genes judged FPKM > 0 by RNAseq. The amount of expression tended to decrease in the order of Ser2P/Ser5P double positive, Ser5P single positive, Ser2P single positive, and Ser2P/Ser5P double negative. Moreover, qPCR confirmed the results for three of the six FPKM = 0 genes (*WDR69*, *SPATA9*, *GGN*), despite their low expression levels (Figure 4C). Generally, RNAseq seems to be more sensitive than ChIPseq for detecting mRNA, because sequence tags of RNAseq concentrate on exons, and more genes could be detected by RNAseq than by ChIPseq (Figure 3A). However, the results shown in Figure 4C indicate that some gene expressions that could not be confirmed with RNAseq were identified with ChIPseq. ChIPseq has the advantage of being able to map RNAPII to an intron or UTR that has few homologs and does not need to consider splicing; therefore, for certain genes, ChIPseq could have higher sensitivity than RNAseq. On the other hand, we also examined whether there was a correlation between the height of the peak of RNAPII and the amount of mRNA (Figure 4D), but no such correlation was found. These data indicated that in terms of the quantification of the amount of mRNA, RNAseq has a much higher sensitivity than ChIPseq.

### RNAPII status can be classified into three categories for transcribed genes

**Figure 5:**
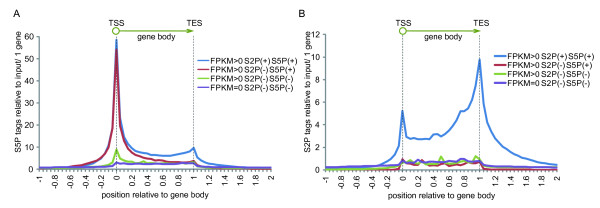
**Relative tags from ChIPSeq indicate three states of RNAPII**. (a) The average Ser5P RNAPII enrichment tags per one gene from ChIPseq data, normalized to input by Peakseq, were summed according to their shifted positions with the definition that gene length was from 0 to 1. Where 0 represents the transcription start site and 1 represents the transcription end site. This indicated three states of RNAPII: Low pausing and low elongation, high pausing and low elongation, and high pausing and high elongation. (b) Ser2P was also analyzed. No significant enrichment was seen, except for Ser2P and Ser5P double positive genes.

The analysis of expression data from RNAseq allowed us to classify genes into Ser2P/Ser5P double positive, single positive, and double negative, according to their different levels of expression. However, this was only for the data from peaks judged to have a P-value < 0.05 and FDR < 0.05 according to ChIPseq, and did not assess the comparison of accumulated amounts of tags in ChIPseq nor tags accumulated in genes outside of this peak. Thus, to re-evaluate the phosphorylation state of RNAPII between these gene categories, we did not utilize the threshold of P-value and FDR and counted tags in each part of the gene body that were covered by Ser2P and/or Ser5P and how frequently they existed in each gene. Figure 5 and Supplementary Figure S3 show in which part of gene the Ser5P-associated tags accumulated (Figure 5A, Additional File 4, Figure S3 A). The data for Ser2P-associated tags are shown in Figure 5B and Supplementary Figure S3 B, which assumes that gene body is from 0 to 1. Considering Ser5P tag counts, although FPKM > 0/Ser2P(-)/Ser5P(+) genes and FPKM > 0/Ser2P(+)/Ser5P(+) genes have similar levels of Ser5P tags around the TSS, of the number of Ser5P tags associated with Ser5P single positive genes decreases around the TES compared with Ser2P/Ser5P double positive. Even if only the existence of Ser5P around the TSS and TES is considered, the classification of our gene groups clearly distinguishes two states of RNAPII, i.e. promoter proximal pausing and active transcription. Moreover, the genes which RNAseq judged FPKM > 0 and ChIPseq judged not to be Ser5P positive, because of the condition of P-value < 0.05 and FDR < 0.05, have Ser5P around the TSS compared with FPKM = 0/Ser2P(-)/Ser5P(-) genes, though at a low level, when the tags are collected and counted.

When tags were summed for genes with FPKM > 0/Ser2P(+)/Ser5P(-), the number of Ser2P tags tended to be high (Additional File 4, Figure S3 B). However, for these genes, the tag count outside of the gene (X axis is more than 1 or less than 0) for Ser2P and Ser5P are also higher than for other gene categories, and this may indicate that they were picked up from the background noise generated by surrounding genes. The Ser5P tags showed a small peak around the TSS of these genes (Additional File 4, Figure S3 A), and when the background was excluded, the shape of the graph obtained from FPKM > 0/Ser2P(+)/Ser5P(-) genes was approximately the same as that from FPKM > 0/Ser2P(-)/Ser5P(-). These results suggest that the genes whose expression is confirmed by RNAseq can be classified into three categories by combining ChIPseq data concerning Ser2P/Ser5P: 'High pausing, High elongation (Ser5P+, Ser2P+)', 'High pausing, Low elongation (Ser5P+, Ser2P-)', and 'Low pausing, Low elongation (Ser5P-, Ser2P-)'.

### Control of mRNA expression is correlated to phased phosphorylation of Ser2 and Ser5

**Figure 6:**
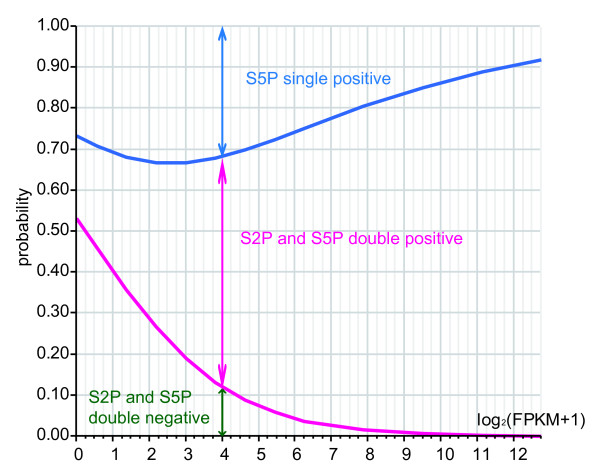
**Nominal logistic fit of RNAPII phosphorylation state by FPKM on log scale**. All FPKM > 0 genes were analyzed (n = 13462). To avoid using the log of 0, we added 1 to each of the FPKM values prior to calculating logs.

The amount of mRNA expression of the genes grouped by phased phosphorylation of Ser2 and Ser5 of RNAPII had a tendency to increase with the level of phosphorylation, leading the predicted three category mode: 'High pausing, High elongation', 'High pausing, Low elongation', and 'Low pausing, Low elongation'. Thus, a gene could be categorized by its FPKM value using logistic regression analysis. 13,462 genes judged to have FPKM values > 0 by RNAseq became the object of the analysis. As mentioned before, false positives could arise by the influence of surrounding genes, and was thought to be about 588 genes (Ser2P single positive); therefore, these genes were counted in this analysis in addition to the Ser2P/Ser5P double negative group. Whole model test and parameter estimates are shown in Table 2. In addition, the phosphorylation status of Ser2 and/or Ser5 of RNAPII was observed for each gene examined according to the FPKM value, and a probability plot was produced (Figure 6). As the value of FPKM obtained by RNAseq rises, the probability that RNAPII is phosphorylated (as judged by ChIPseq; P-value < 0.05, FDR < 0.05) rises. Finally, half of the genes with an FPKM value of about 0 belong to the 'Low pausing, Low elongation' group, in which the existence of phosphorylated RNAPII cannot be proven. However, among the genes for which the existence of phosphorylated RNAPII could be proven, more than half of the genes for which the FPKM value was one or less were Ser5P single positive. The probability of Ser5P single positive genes increases until their values of FPKM rise to about 6 (Figure 6). It can be said that the genes whose FPKM is relatively high have an unexpected high possibility of being judged as 'pausing' genes. The number of genes associated with Ser2P/Ser5P double positive RNAPII increased with increasing FPKM value, finally reaching 90 percent or more. Our analysis presumes a qualitative value, like the phosphorylation of Ser2 and Ser5, from a various amounts of gene expression (FPKM). Although each group's borderline, which inclines sideways in the probability plot of the logistic regression analysis, indicates the existence of another factor, it seems that the phosphorylation of RNAPII correlates with the gene expression level. This is the first model of various mRNA expressions using epigenetic factors. Ultimately, the amount of mRNA expression could be explained using a similar model in combination with other epigenetic factors, such as transcription factors and histone modification.

**Table 2:** Logistic regression analysis for RNAPII CTD phosphorylation Whole model test

Model	Log likelihood		DF	Chi-square	Prob > ChiSq	
Difference	1132.762		2	2265.525	< 0.0001	
Full	12914.338					
Reduced	14047.1					
Rsquare (U)	0.0806					
Observations (or sum weights)	13462					

**Parameter estimates**						

Term	Estimate	Std Error	Chi-square	Prob > ChiSq	Lower CI (95%)	Upper CI (95%)

Intercept [S2P- S5P-]	0.6877	0.0445	238.89	< 0.0001	0.6005	0.7749
log2(FPKM+1) [S2P- S5P-]	-0.4149	0.0158	691.98	< 0.0001	-0.4458	-0.384
Intercept [S2P+ S5P+]	-0.2791	0.0412	45.89	< 0.0001	-0.3599	-0.1984
log2(FPKM+1) [S2P+ S5P+]	0.2127	0.0105	410.32	< 0.0001	0.1921	0.2333

For log odds of [S2P-S5P-]/[S2P-S5P+], [S2P+S5P+]/[S2P-S5P+]

## Conclusions

We studied the association between mRNA expression level and RNAPII phosphorylation state in Hela cells using a deep sequencer for RNAseq and ChIPseq analysis. During verification to improve the accuracy of RNAseq, we found that the correlation between RNAseq and past expression microarray data could be increased by adjusting the 'Max multihits' parameter. We optimized this parameter such that it minimized the risk of reading genes that are not simultaneously expressed. We also produced an antibody against the phosphorylated form of RNAPII, which allowed the genome-wide visualization of the state of RNAPII phosphorylation using ChIPseq. RNAseq and ChIPseq showed a very high correlation, and the existence of RNAPII on approximately 82% of genes that were detected with RNAseq was confirmed in ChIPseq. In addition, when we examined the relationship between the phosphorylation state of RNAPII and the level of mRNA expression, phosphorylation of both Ser2 and Ser5 of RNAPII was confirmed for almost all highly expressing genes. When only Ser5 of PNAPII was phosphorylated, low mRNA expression was detectable by RNAseq, in spite of the pausing state. Moreover, when tag counts of Ser5P were counted for genes identified only with RNAseq, the existence of a slightly higher level of Ser5P was detected compared with the negative control. This indicated that transcriptional adjustment is performed in two stages: promoter escape and active elongation. We also provide a hypothesis that gene expression can be classified into three groups according to the phosphorylation state of RNAPII.

## Methods

### Cell culture

Hela cells were cultured in Dulbecco's modified Eagle's medium (DMEM) supplemented with 10% fetal bovine serum under a humidified atmosphere with 5% CO_2 _at 37°C.

### Production of rat monoclonal antibodies

The RNA polymerase II (RNAPII) Ser2P and Ser5P antigens were synthesized based on their specific sequences, Ser2P: SPTSPSYSphPTSPSYSPTSPS and Ser5P: SPTSPSYSPTSphPSYSPTSPS (Sigma-Aldrich). A C-terminal cysteine residue that was not part of the sequence was introduced to allow coupling to the carrier protein maleimide-activated keyhole limpet hemocyanin (Thermo Scientific). The coupling reaction was performed according to the supplier's instructions. Rat monoclonal antibodies were generated based on the rat lymph node method established by Sado et al. [29]. A 10-week-old female lzm rat (Japan SLC) was injected in the rear footpads with 500 μl of an emulsion containing 200 μg RNAP2 Ser2P or Ser5P peptide and Freund's complete adjuvant. After two weeks, the cells from the lymph nodes of the immunized rat were fused with mouse myeloma Sp2/0-Ag14 cells at a ratio of 5:1 in 50% polyethylene glycol (Merck) solution. The resulting hybridoma cells were plated onto 96-well plates and cultured in HAT selection medium [hybridoma SFM medium (Invitrogen); 10% fetal bovine serum; 10% BM-Condimed H1 (Roche); 100 μM hypoxanthine; 0.4 μM aminopterin; 1.6 μM thymidine]. At seven days post-fusion, the hybridoma supernatants were screened using an enzyme-linked immunosorbent assay (ELISA) against each antigen. Positive clones were subcloned and rescreened by ELISA (Table 1). To prepare hybridoma supernatants containing highly concentrated antibodies, the resulting positive clones, 3E7C7 for RNAP2 Ser2P and 1H4B6 for RNAP2 Ser5P, were cultured at a high cell density using a MiniPERM bioreactor (Vivascience).

### ELISA

BSA conjugated RNAPII Ser2P or Ser5P peptides (5 μg/mL) at dilutions ranging from 1:100 to 1:100000 in ELISA buffer [10 mM sodium phosphate pH7.0] were adsorbed on the surface of 96-well costar Serocluster 96 Well "U" Bottom Plates (Corning) by overnight incubation at 4°C. To avoid non-specific binding, the plates were blocked with 1% bovine serum albumin (BSA) in PBS. Hybridoma supernatants were applied to the plates and incubated for 1 h at room temperature and then washed three times with PBS. The plates were incubated for 30 min at room temperature with alkaline phosphatase-conjugated anti-rat IgG antibody (Sigma) at a dilution of 1:10000. After washing three times with TBS-T, immunoreactivity was visualized using a pNPP phosphatase substrate system (KPL).

### Immunoblotting

Hela cells were washed twice with phosphate buffered saline (PBS), centrifuged, and then resuspended in 2 × SDS sample buffer. The samples were separated by SDS-PAGE and transferred to a nitrocellulose membrane with iBlot (Invitrogen). The membrane was blocked for 1 h in 5% (w/v) skimmed milk in Tris-buffered saline containing 0.05% (v/v) Tween 20 (TBST), then incubated with primary antibodies in solution 1 (TOYOBO). The blot was then incubated with horseradish peroxidase-labeled secondary antibodies and detected using the WestDura chemiluminescence kit (Pierce). The primary antibodies were anti-RNAPII Ser2P (3E7C7, hybridoma supernatant, 1:1000; Figure 2B and Table 1), anti-RNAPII Ser5P (1H4B6, hybridoma supernatant, 1:1000; Figure 2B and Table 1), and sc-899, the antibody against the N-terminus of RNAPII (1:1000; Figure 2B). Secondary antibodies were horseradish peroxidase-conjugated anti-rat IgG antibodies (1:5000; GE Healthcare).

### Quantitative RT-PCR

Total RNA was isolated and reversed-transcribed with Takara Prime Script Reverse Transcriptase and an oligo dT primer, as previously described [30]. Quantitative-PCR (Q-PCR) was performed using TaKaRa SYBR Premix Dimer Eraser. Q-PCR data are presented as the mean ± standard deviation of three independent experiments. Primer sequences are available upon request.

### RNASeq

Libraries were generated by the modified Illumina protocol using the mRNAseq preparation kit. Briefly, 1 μg of total RNA was enriched for polyA RNA by two successive rounds of oligo(dT) selection. The polyA RNA was then fragmented, and first-strand cDNA synthesis was performed using random hexamer priming. Following second-strand cDNA synthesis, dsDNA was repaired using T4 DNA polymerase, Klenow enzyme, and T4 polynucleotide kinase (PNK) (New England Biolabs), followed by treatment with Klenow exo^- ^to add an A base to the 3' end. After ligation of the Solexa adaptor using TaKaRa ligation Mix (TaKaRa), the adaptor-ligated DNAs were amplified using Solexa PCR primers for 18 cycles, and the amplified library was isolated from an agarose gel. The samples were purified using the QIAquick MinElute kit (Qiagen) at each preparation step.

### RNASeq data analysis

For each sample, cDNA was sequenced (single 36 bp read) by an Illumina Genome Analyzer GAIIx. The base-called sequences were obtained using SCS2.7 from RNAseq image data. To calculate the total amount of the transcripts of each mRNA, a series of programs-Bowtie [31], TopHat (v1.1.4) [18], and Cufflinks (v0.9.3) [19]-were used. Briefly, RNAseq reads were mapped against the whole reference genome (hg19) using Bowtie. The reads that did not align to the genome but were mapped to potential splice junctions by TopHat were considered to bridge splice junctions. The quantification of transcripts, with normalization for gene length, was performed by Cufflinks. All of the parameters, except 'Max multihits' (TopHat), were substituted with default options (TopHat: -g options as utilized as "multihits". Cufflinks: default suggested as -m 230 -s 20 -I 300000). The 'Max multihits' was set at 1, 2, 5, 10, 20, 40, 100, and 1000, and then the number of FPKM > 0 genes was determined (Figure 1A, Additional File 2, Figure S2). The Spearman's correlation coefficients with microarray data (Figure 1B), the percentage of splice sites that were included in the gene body (Figure 1C), and histograms of FPKM distributions at three 'Max multihits' values (Figure 1D) were plotted.

### Gene ontology and Fishers' exact test

For the analysis, we used Funcassociate 2 [32], which is a web application tool http://llama.mshri.on.ca/funcassociate/ that finds significant Gene ontology terms from large-scale experimentation. All of the parameters were substituted with default options, i.e. Mode:unordered, Over/Under:over, Simulations:1000, and Significance Cutoff:0.05.

### Chromatin Immunoprecipitation (ChIP)

ChIP assays were performed by modifying the Upstate Biotechnology protocol, as described previously [33] except adding 40 mM β-glycerophosphate and 1 mM sodium fluoride to immunoprecipitation buffer, utilizing rat monoclonal antibodies against RNAPII Ser2P (3E7C7, 5 μg; Figure 2B and Table 1) and RNAPII Ser2P (3E7C7, 5 μg; Figure 2B and Table 1). Relative recruitment (Figure 3C) was defined as the ratio of amplification of the PCR product relative to 1% of input genomic DNA. Q-PCR data are presented as the mean ± standard deviation of three independent experiments. We designed PCR primers for gene regions within the 3 kb downstream of the 5'-start of each gene and within the 3 kb upstream of the 3'-end of each gene because Ser5 phosphorylated RNAPII is positioned in the coding region at +2 to +4 kb from start site, as well as upstream of start site [12]. Coding regions were used to prevent any effects from neighboring genes. Primer sequences are available upon request.

### ChIPSeq

For ChIPseq, sample preparation was performed using the ChIP protocol described above. The ChIP DNA and the Input DNA ends were repaired using T4 DNA polymerase, Klenow enzyme, and T4 polynucleotide kinase (PNK) (New England Biolabs), followed by treatment with Klenow exo^- ^to add an A base to the 3' end. After ligation of the Solexa adaptor using TaKaRa ligation Mix (TaKaRa), the adaptor-ligated DNAs were amplified using Solexa PCR primers for 18 cycles, and the amplified library was isolated from an agarose gel. The samples were purified using the QIAquick MinElute kit (Qiagen) at each preparation step. The purified library was used for cluster generation and sequencing analysis using the Genome Analyzer GAIIx (Illumina K. K.).

### ChIPSeq data analysis

Base-called sequences were obtained using SCS2.7 from ChIPseq image data. The sequence tags for RNAPII Ser2P and Ser5P and Input were aligned to the human genome (hg19) using Bowtie [31] software. Peak detection and identification of binding sites of RNAPII Ser2P and Ser5P were obtained by correcting from Input DNA using Peakseq software, as described previously [24]. The box plot of RNAPII Ser2P and Ser5P enriched regions that were found in Peakseq when using the threshold of P-value < 0.05, Q-value < 0.05 is shown in Figure 3B. We defined RNAPII recruitment as positive if the box plot overlapped the gene body to create the Venn diagram (Figure 3A, Additional File 2, Figure S2). For the detection of the binding site of RNAPII Ser2P and Ser5P, all tags normalized to input by Peakseq were summed according to their shifted positions, with the definition that a gene length was 1, and along the horizontal axis. 0 indicates the TSS (Transcription start site) and 1 indicates the TES (Transcription end site) (Figure 2C, 5 and Additional File 4, Figure S3).

Any experimental procedure in this study does not contain any animal experiment.

### Data availability

The raw illumina sequencing data are available from the DNA Data Bank of Japan (DDBJ) with accession number [DDBJ: DRA000363].

### Logistic regression analysis

Logistic regression was used to estimate the probability of each RNAPII CTP phosphorylation state across the FPKM range. A logistic probability plot for RNAPII state was also created. All calculations were performed using JMP v 8.0.2 (SAS, Cary, NC) running under Windows XP.

## List of abbreviations

ChIP: chromatin immunoprecipitation; RNAPII: RNA polymerase II; CTD: C-terminal domain; TSS: transcription start site; TES: transcription end site; TFIIH: Transcription factor II H; NELF: Negative elongation factor; DSIF: DRB sensitivity including factor

## Authors' contributions

JO, AH, and YO designed the experiments. JO performed the experiments. TY and TT generated anti-Ser2P and Ser5P monoclonal antibodies. JO, KM, and YO analyzed the data. JO and YO wrote the manuscript. SO, KA, and YO edited the manuscript. All authors have read and approved the manuscript.

## Supplementary Material

Additional file 1 **Supplementary Figure S1**. mRNA expressions of representative genes whose FPKM values were low. Their expressions could be confirmed by PCR. We chose the low FPKM value genes at random from the group in which the existence of phosphorylated RNAPII could not be confirmed by ChIPseq. Click here for file

Additional file 2 **Supplementary Figure S2**. (a) Venn diagram summarizing the overlap between FPKM > 0 genes, Ser2P genes, and Ser5P genes according the 'Max multihits' parameter. (b) A line graph showing how many detected genes increase in each category when 'Max multihits' parameter increases from 1. Both (a) and (b) indicated that when 'Max multihits' parameter increases, the number of genes detected by RNAseq rises, mainly in the group RNAseq(+), Ser2P(-), Ser5P(-). Click here for file

Additional file 3 **Supplementary Table S1**. Significant GO terms for each category. To investigate the functional relationship among pausing/active genes and gene functions, we analyzed significant association using Gene ontology and Fishers' exact test. Though hundreds of GO terms were judged to be significant for double positive (RNAseq+/-, Ser2P+m Ser5P+) genes, for the other categories, significant GO terms were merely found. Click here for file

Additional file 4 **Supplementary Figure S3**. Relative tags from FPKM > 0/Ser2P(+)/Ser5P(-) genes in ChIPseq indicate their source as background noise. When Ser5P (a) and Ser2P (b) tags were summed for genes with FPKM > 0/Ser2P(+)/Ser5P(-), the tag count outside of the gene was higher than for other gene categories. This may indicate that they were picked up from the background noise generated by surrounding genes. Click here for file
