# Supplementary figures and images for: The classification of mRNA expression levels by the phosphorylation state of RNAPII CTD based on a combined genome-wide approach
Source: BMC Genomics. 2011 Oct 20;12:516. doi: 10.1186/1471-2164-12-516 (PMC3209707; doi:10.1186/1471-2164-12-516)

|                  | FPKM | Ser2P | Ser5P | RT(+)                                                                               |                                                                                     |                                                                                     | RT(-) |  |  |
|------------------|------|-------|-------|-------------------------------------------------------------------------------------|-------------------------------------------------------------------------------------|-------------------------------------------------------------------------------------|-------|--|--|
| <i>CYP2S1</i>    | 1.97 | (-)   | (-)   | 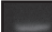 | 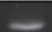 | 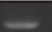 |       |  |  |
| <i>C17orf108</i> | 0.56 | (-)   | (-)   | 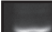 | 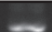 | 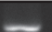 |       |  |  |
| <i>SYPL2</i>     | 0.40 | (-)   | (-)   | 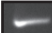 | 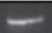 | 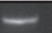 |       |  |  |
| <i>KIF5C</i>     | 0.10 | (-)   | (-)   | 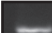 | 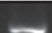 | 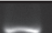 |       |  |  |
| <i>FAT2</i>      | 0.05 | (-)   | (-)   | 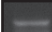 | 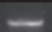 | 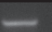 |       |  |  |

Supplement: Additional file 1 — Supplementary Figure S1. mRNA expressions of representative genes whose FPKM values were low. Their expressions could be confirmed by PCR. We chose the low FPKM value genes at random from the group in which the existence of phosphorylated RNAPII could not be confirmed by ChIPseq. [file 1471-2164-12-516-S1.PDF]

A

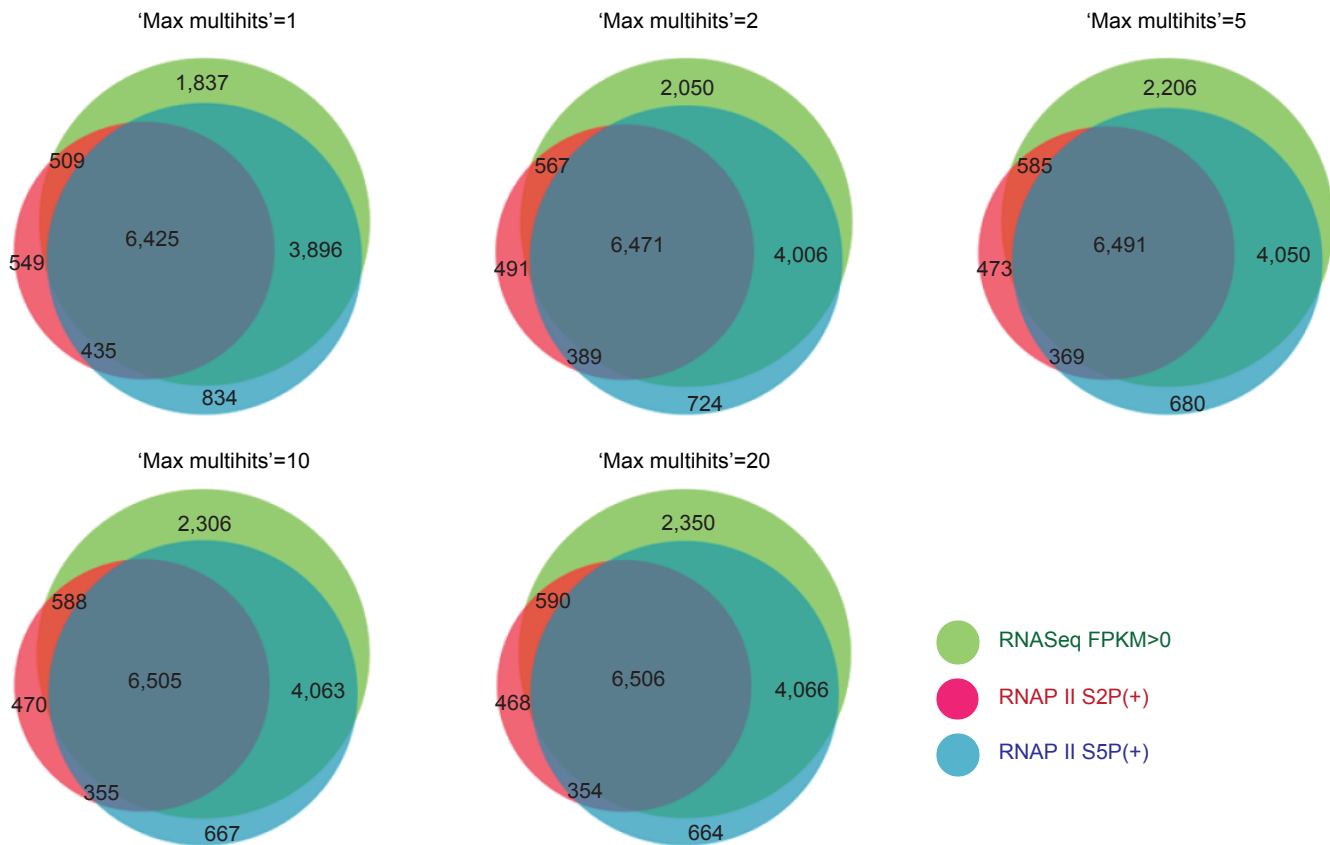

B

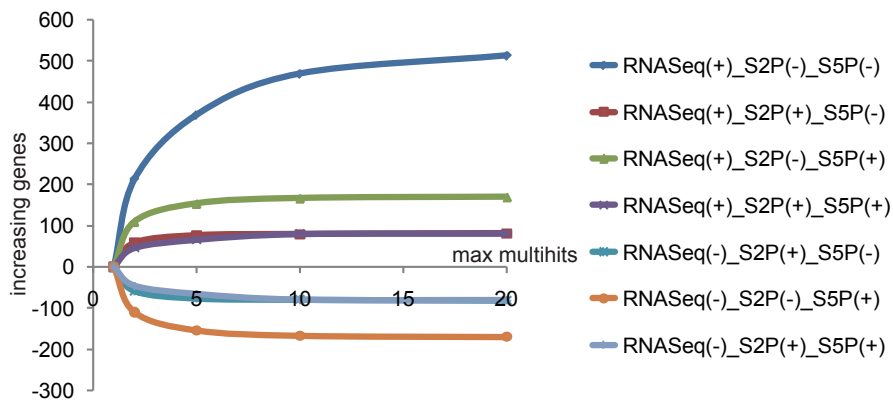

Supplement: Additional file 2 — Supplementary Figure S2. (a) Venn diagram summarizing the overlap between FPKM > 0 genes, Ser2P genes, and Ser5P genes according the 'Max multihits' parameter. (b) A line graph showing how many detected genes increase in each category when 'Max multihits' parameter increases from 1. Both (a) and (b) indicated that when 'Max multihits' parameter increases, the number of genes detected by RNAseq rises, mainly in the group RNAseq(+), Ser2P(-), Ser5P(-). [file 1471-2164-12-516-S2.PDF]

A

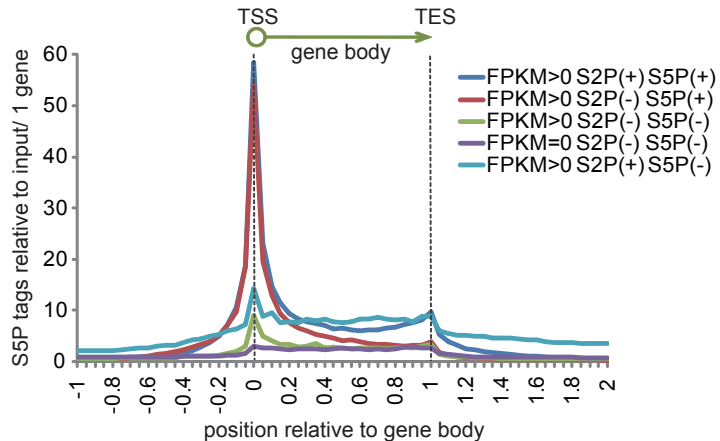

B

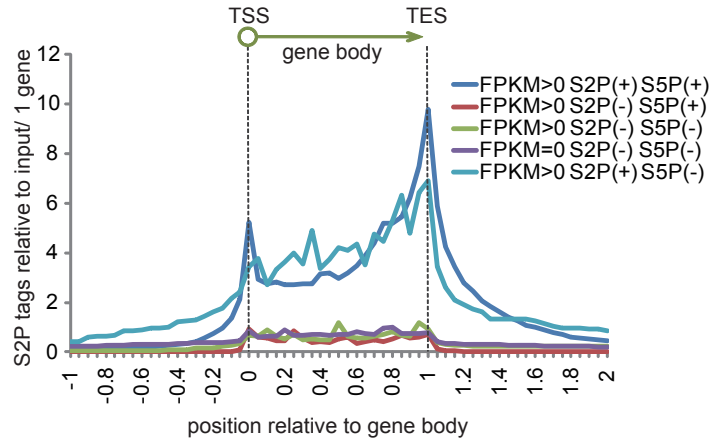

Supplement: Additional file 4 — Supplementary Figure S3. Relative tags from FPKM > 0/Ser2P(+)/Ser5P(-) genes in ChIPseq indicate their source as background noise. When Ser5P (a) and Ser2P (b) tags were summed for genes with FPKM > 0/Ser2P(+)/Ser5P(-), the tag count outside of the gene was higher than for other gene categories. This may indicate that they were picked up from the background noise generated by surrounding genes. [file 1471-2164-12-516-S4.PDF]
